# Online database for brain cancer-implicated genes: exploring the subtype-specific mechanisms of brain cancer

**DOI:** 10.1186/s12864-021-07793-x

**Published:** 2021-06-18

**Authors:** Min Zhao, Yining Liu, Guiqiong Ding, Dacheng Qu, Hong Qu

**Affiliations:** 1 School of Science, Technology and Engineering, University of the Sunshine Coast, Maroochydore DC, Sippy Downs, Queensland 4558 Australia; 2 The School of Public Health, Institute for Chemical Carcinogenesis, Guangzhou Medical University, Guangzhou, China; 3 School of Computer Science & Technology, Beijing Institute of Technology, Beijing, 100081 China; 4 Information Center, China Association for Science and Technology, Beijing, 100863 China; 5 Center for Bioinformatics, State Key Laboratory of Protein and Plant Gene Research, College of Life Sciences, Peking University, Beijing, 100871 P.R. China

**Keywords:** Brain cancer, Database, Genetic, Subtype, Systems biology, Bioinformatics

## Abstract

**Background:**

Brain cancer is one of the eight most common cancers occurring in people aged 40+ and is the fifth-leading cause of cancer-related deaths for males aged 40–59. Accurate subtype identification is crucial for precise therapeutic treatment, which largely depends on understanding the biological pathways and regulatory mechanisms associated with different brain cancer subtypes. Unfortunately, the subtype-implicated genes that have been identified are scattered in thousands of published studies. So, systematic literature curation and cross-validation could provide a solid base for comparative genetic studies about major subtypes.

**Results:**

Here, we constructed a literature-based brain cancer gene database (BCGene). In the current release, we have a collection of 1421 unique human genes gathered through an extensive manual examination of over 6000 PubMed abstracts. We comprehensively annotated those curated genes to facilitate biological pathway identification, cancer genomic comparison, and differential expression analysis in various anatomical brain regions. By curating cancer subtypes from the literature, our database provides a basis for exploring the common and unique genetic mechanisms among 40 brain cancer subtypes. By further prioritizing the relative importance of those curated genes in the development of brain cancer, we identified 33 top-ranked genes with evidence mentioned only once in the literature, which were significantly associated with survival rates in a combined dataset of 2997 brain cancer cases.

**Conclusion:**

BCGene provides a useful tool for exploring the genetic mechanisms of and gene priorities in brain cancer. BCGene is freely available to academic users at http://soft.bioinfo-minzhao.org/bcgene/.

**Supplementary Information:**

The online version contains supplementary material available at 10.1186/s12864-021-07793-x.

## Background

Brain cancer, a leading type of cancer that causes death in both children and adults, was diagnosed in about 300,000 new cases and caused 241,000 deaths globally in 2018 [1]. More recently, mortality figures of brain and other nervous system cancers in the United States caused an estimated 23,890 deaths in 2020 (12,590 males and 10,300 females) [2]. As a heterogeneous disease, uncontrolled cell growth in brain cancer has complex molecular mechanisms, which may be caused by promoter methylation, deregulated gene expression, and/or genetically altered tumor-suppressor genes and oncogenes [3, 4]. According to the most recent data summary in the cancer genomics data portal cBioPortal, there are 6166 cases covering a comprehensive multi-omics data of genetic alterations and deregulated expression. Although those genomic profilings play a major role in shaping the genetics and transcriptome of brain tumours, the literature-based genetic differences of various brain cancers are still largely unknown.

Histologically, glioma is the most common tumor type and includes astrocytoma, ependymoma, and oligodendroglioma. Oligodendroglioma is more sensitive to chemotherapy than is astrocytoma, and therefore has a better overall prognosis [5]. The overall 5-year survival rate of brain cancer patients is approximately 36%, but the 5-year survival rate of oligodendroglioma patients is about 80.6%, and the 10-year relative survival rate is 63.8%. However, the 5-year survival rate for patients with glioblastoma (also known as glioblastoma multiforme, or GBM) is only 5.4%, and the 10-year survival rate is only 2.7% [6]. Therefore, exact identification of glioma subtypes is essential for neuro-oncologists to provide the best treatment. Although many existing clinical and histological methods identify brain cancer subtypes, molecular subtype information can independently and reliably confirm or refute those identifications, thus providing more accurate diagnostic evidence.

Although thousands of published articles have focus on brain cancer, a literature-based effort that scrutinizes both the common and unique genetic information of each brain cancer subtype does not exist. Additionally, most functional or clinical studies have been single-gene–based, and thus have failed to provide any descriptions of tumorigenesis for different cancer subtypes. We hypothesize that mapping literature-based information to public cancer genomics data will provide a more comprehensive genetic perspective for brain cancer and those important subtypes. Therefore, we developed a database, BCGene, that is a reusable genetic resource for brain cancer, has links to the appropriate literature, and provides global genetic profiles of brain cancer subtypes. The curated genes in the literature can be prioritized according to their correlations with brain cancer, and common and unique cellular events in different brain cancer subtypes can be identified.

## Materials and methods

### Literature search and curation

**Fig. 1:**
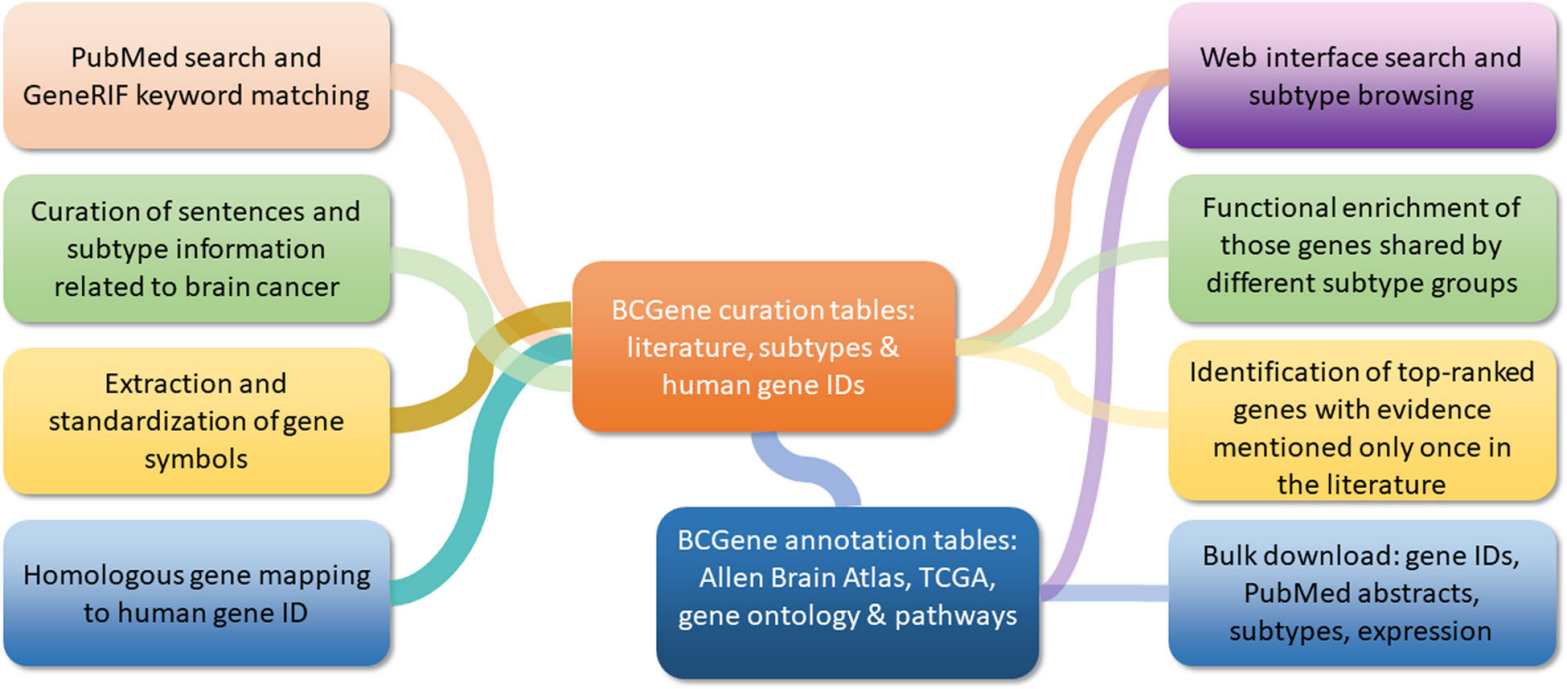
The flowchart for brain cancer gene collection, database construction and gene function analysis

As shown in the flowchart in Fig. 1, we relied heavily on the PubMed and GeneRIF (Gene Reference Into Function) databases to assemble our collection of brain cancer-implicated genes [7]. Specifically, in the GeneRIF database, we performed a keyword-based query using a Perl regular expression to extract relevant sentences we had previously described [8]: “[gG] liomas or [gG] lioblastomas or [Bb] rain tumor or [Bb] rain cancer or [Aa] strocytomas or [Oo] ligodendrogliomas or [Ee] pendymomas or [Mm] eningiomas or [Hh] aemangioblastomas or [Aa] coustic neuromas or [Cc] raniopharyngiomas or [Ll] ymphomas or [Hh] aemangiopericytomas or [Ss] pinal cord tumor or [Nn] euroectodermal tumor or [Mm] edulloblastoma or [Pp] ituitary tumor”. In total, within 2881 unique PubMed abstracts, we found 9304 short sentences related to brain cancer. We used the same expression to search the PubMed database, and all matching records from PubMed and GeneRIF were merged to remove redundancies. Further literature curation included clustering abstracts, extracting matching cancer subtypes, collecting species information, and formalizing gene symbols. For example, in the sentence “re-expression of N-cadherin in gliomas restores cell polarity and strongly reduces cell velocity, suggesting that loss of N-cadherin could contribute to the invasive capacity of tumour astrocytes”, N-cadherin is a common alias for the gene *CDH2* in the Human Gene Nomenclature Database. We also collected tumor subtypes, such as “gliomas”. For non-human genes, we mapped all genes to human orthologous genes. In total, we curated 1421 human protein-coding genes (Table S1).

### Biological annotation and pre-calculated data

To provide biological insight for those collected genes, we retrieved comprehensive biological functional annotations from public resources as described previously [9]. In addition, we used The Cancer Genome Atlas (TCGA) large-scale database to calculate genomic mutation information. For example, the resulting copy number gains and losses in TCGA-GBM and TCGA low-grade glioma (LGG) will enable investigation of changes at the thousands-of-bases level, which may have been overlooked by those published studies focusing on the single nucleotide mutations. We also mapped our 1421 genes to the gene expression information from all brain regions in the most updated Allen Human Brain Atlas, thus providing potential gene expression patterns for hundreds of anatomical locations.

### The web interface

Based on a systematic survey of genes implicated in brain cancer in the literature, we developed a web interface to make those annotations publicly available. From our web interface, curated subtype information allows users to explore all brain cancer-implicated genes, and the amount of literature evidence for each gene provides a guide to how reliably a gene of interest is associated with brain cancer. We also built a responsive, mobile-friendly webpage by using a Bootstrap framework to provide a grid-based layout.

**Fig. 2:**
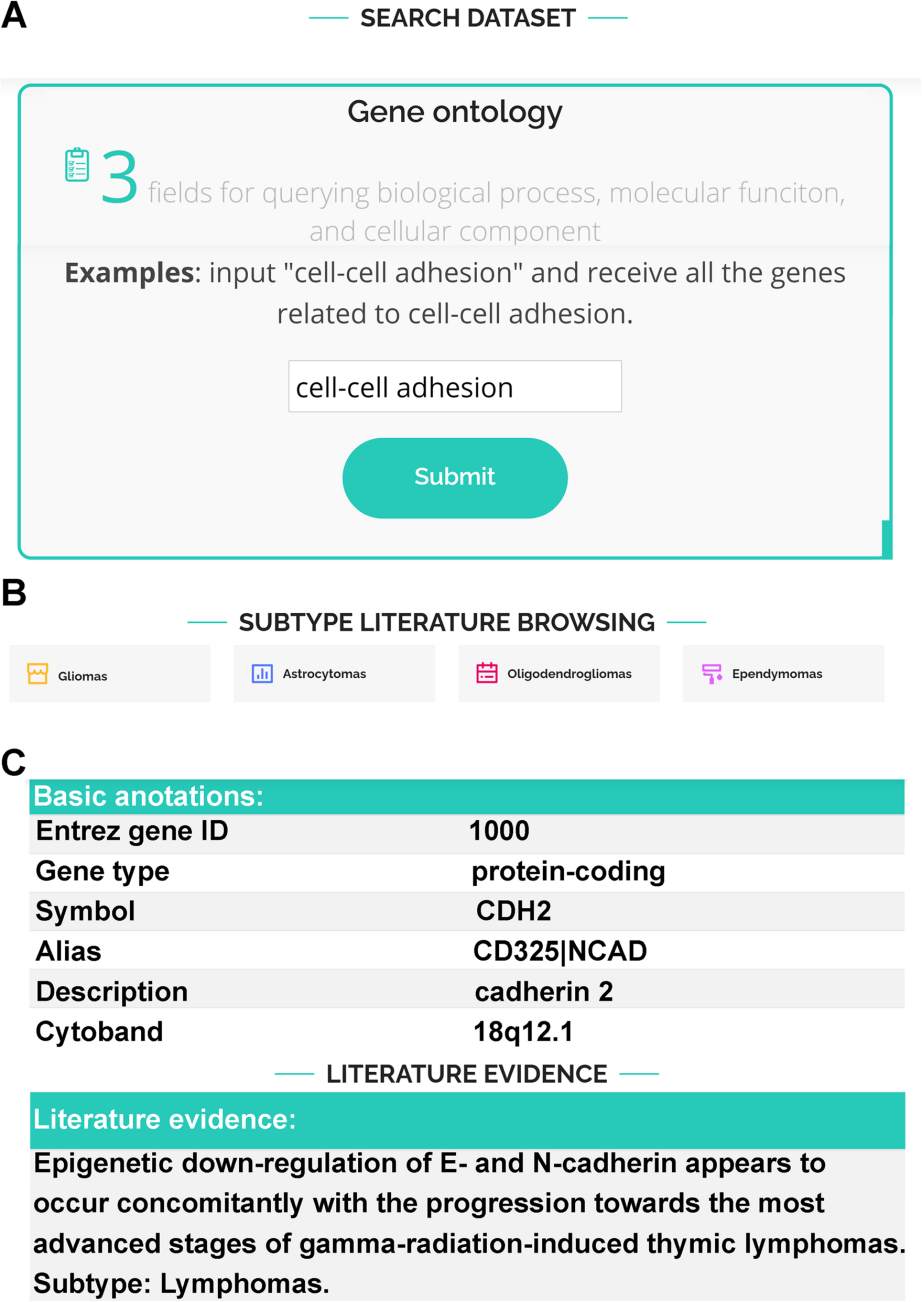
The BCGene database web interface. **A** Keyword-based query interface. **B** Browsing genes and literature using cancer subtypes. **C** Basic annotations and associated literature mentioning human genes in BCGene

As shown in Fig. 2A, three search modules are implemented by entering 1) a gene name or its description; 2) a gene ontology, (including biological processes), molecular function, and cellular component; and 3) any keywords of interest in the curated literature. These keyword-based queries enables users to identify both curated genes and the related literature on a specific biological topic. For advanced bioinformatics analysis, users may download curated genes, applicable literature, and subtypes in bulk (Fig. 2B). To organize information for each gene, we divided our annotation details into six categories: gene information, published evidence, gene ontology, biochemical pathway [10], genetic mutation summary from TCGA, and gene expression information from the Allen Brain Map (Fig. 2C).

### Functional enrichment analysis

We used ToppFun [11] to conduct a functional enrichment analysis of the 44 genes shared by multiple subtype groups. In that analysis, we used all 1421 genes in our BCGene database as background and then used the hypergeometric model, comparing the differences between the 44 annotated genes and all 1421 genes, to identify the statistical significances of enriched annotations. Since we calculated thousands of raw *p*-values, we then used the Benjamini-Hochberg multiple correction method to adjust those raw values. Focusing on the most significant changes, we extracted the enriched annotations with corrected *p*-values less than 0.01 and used them as over-representative annotations for the 44 genes. Finally, we visualized those enriched biological process terms by the TreeMap package using R language.

### Gene prioritization based on functional similarity

Since we have 883 genes with only a single study in the literature, we had to consider the relative importance of each gene when ranking candidate genes according to their functions. To accomplish this, we first built a gold standard, brain cancer gene list that we subsequently used to train an algorithm to identify important functional features. The training gene list included the 27 most reliable genes, each of which was supported by 20 or more published studies in the literature. To prioritize the relative importance based on functional similarity, we first used the gene ranking tool ToppGene [11] to generate a functional matrix of our 27 training genes based on 12 features including three namespaces from gene ontology, human phenotype ontology, protein domains, gene family, biological pathways [10], known protein-protein interactions, binding transcription factors, co-expression patterns, disease annotations, and data mined from the literature. Then we calculated the similarity score to the functional matrix for each of the 12 features. For a test gene with lack of annotations, the similarity score was set to − 1. Otherwise, the value of the similarity score was between 0 and 1. The derived 12 similarity scores of each test gene were summarized into an overall similarity score based on statistical meta-analysis.

### Cancer genomic analysis of the 33 top-ranked genes that are mentioned in only one published article

We input the 33 genes that have only one published study into cBioPortal to obtain a summary pattern across multiple brain cancer datasets [12]. Then, using the OncoPrint module in cBioPortal, we visualized the sample-based mutational patterns of 2997 brain cancer samples from 14 studies. To provide the most comprehensive mutational profile, we included the most important genetic mutations in cancer development and progression: single nucleotide variations, gene fusions, and copy number variations (CNVs) [13–15]. We also used mutually exclusive analyses as an overview for mutational complementary patterns across all the samples. Finally, we plotted the correlations between mRNA expression and copy number variant/methylation for each gene of interest and conducted an overall survival analysis of the 2997 patient samples found with at least one of those 33 genes.

## Results and discussion

### The literature frequency for various brain cancer subtypes

**Fig. 3:**
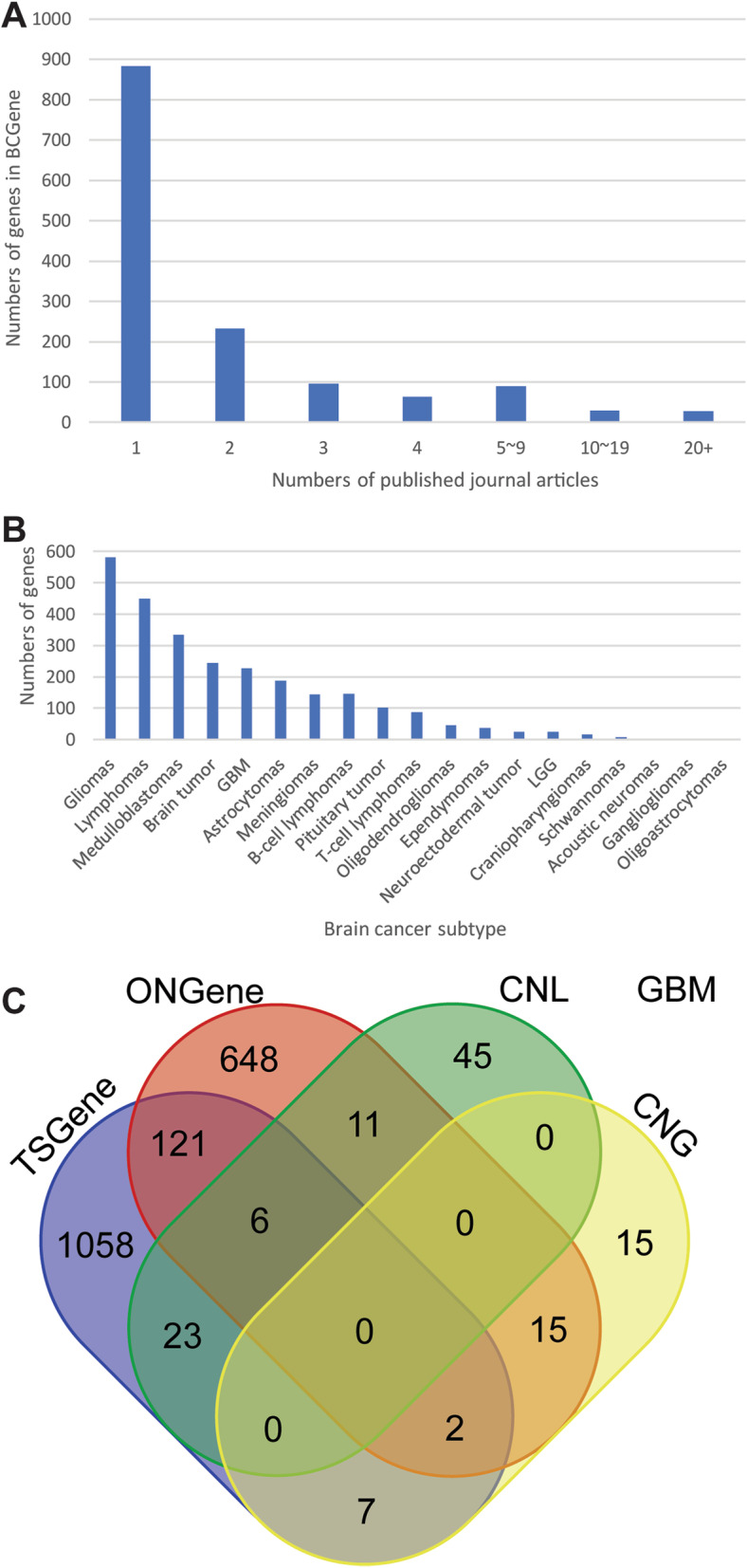
Overall statistics. **A** The distribution of the numbers of published articles related to all brain cancer genes in the database. **B** The numbers of genes in each subtype. **C** Venn diagram of the numbers of potential tumor suppressors (TSGene) and oncogenes (ONGene) for glioblastoma (GBM). CNL, copy number loss; CNG, copy number gain

Based on our comprehensive literature curation, we cleaned up all the associations between brain cancer genes and the literature before conducting further analyses. As shown in Fig. 3A, we found 27 genes that were each supported by more than 20 PubMed abstracts. However, 883 of the 1421 genes implicated in brain cancer (62%) were supported by only a single evidentiary mention in the literature; so obviously, those genes’ functions need further experimental validation. Using cancer subtype keywords, we assigned the 1421 genes to different subtypes, while a gene could be associated with multiple cancer subtypes, each subtype has its own literature-based evidence (Table S2). As shown in Fig. 3B, the top three keywords were: glioma (associated with 582 genes), lymphoma (associated with 450 genes), and medulloblastoma (associated with 245 genes). To explore the genetic heterogeneity of brain cancer, we grouped curated subtype information. For example, astrocytoma, oligodendroglioma, ependymoma, GBM, LGG, ganglioglioma, and oligoastrocytoma were all grouped as gliomas, and medulloblastoma was grouped with neuroectodermal tumors. Then, we subsequently identified 809 glioma-related genes and 354 neuroectodermal tumor-related genes in those two major subtype groups.

After we curated 227 and 25 genes for GBM and LGG, respectively, we summarized all the GBM and LGG CNVs on the gene pages in BCGene. To demonstrate how well our data identifies potential tumor suppressors and oncogenes, we first identified 85 GBM-associated tumor suppressors with more copy number loss (the ratio between copy number loss and copy number gain > 2.0) and 39 GBM-associated oncogenes with more copy number gain (the ratio between copy number gain and copy number loss > 2.0). Then, by cross mapping to the tumor suppressor and oncogene databases (TSGene 2.0 [16] and ONGene [8], respectively) (Fig. 3C), we found that 23 GBM genes with more frequent copy number loss are known tumor suppressor genes, and another 15 GBM genes with more frequent copy number gain are known oncogenes.

### Functional enrichment of those genes shared by different subtype groups

**Fig. 4:**
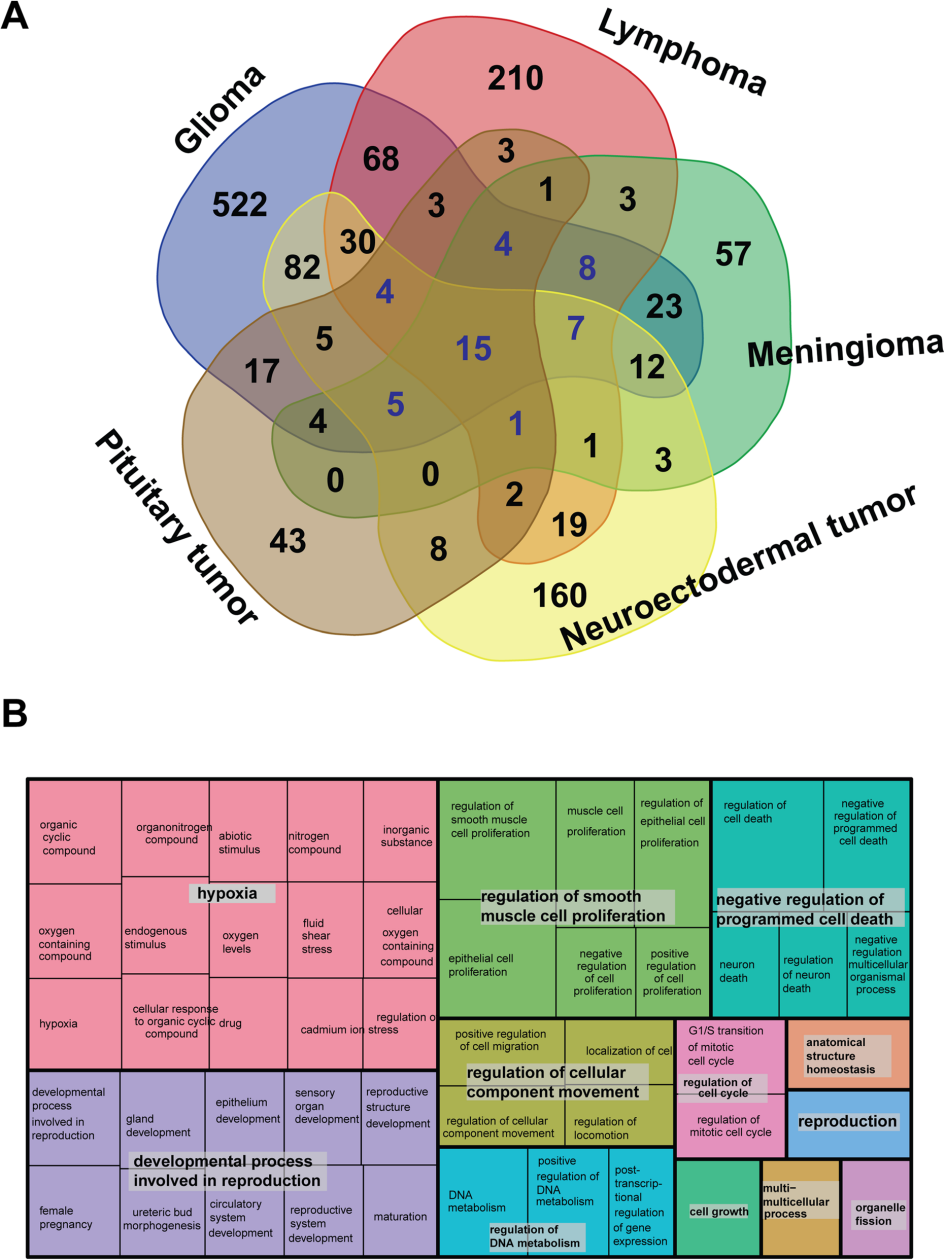
Overlapping and functional enrichment for genes associated with different subtypes. **A** Venn diagram of known genes from different subtypes. **B** Gene ontology enrichment analysis of the 44 genes shared by multiple subtypes

To check the genetic heterogeneity of the high-level cancer subtype groups, we overlapped their associated genes to compare the common and unique genetic features of the five subtype groups (glioma, lymphoma, meningioma, neuroectodermal tumor, and pituitary tumor) (Fig. 4A) and found 44 genes belonging to four or more groups. Gene ontology enrichment analysis revealed that those 44 genes are highly associated with 12 functional categories (Fig. 4B). Some of those categories are highly related to cancer, such as negative regulation of programmed cell death (Benjamini and Hochberg false discovery rate (FDR) corrected *p*-value = 4.35E-05), DNA metabolism regulation (Benjamini and Hochberg FDR corrected *p*-value = 1.42E-04), and regulation of the mitotic G1/S transition (Benjamini and Hochberg FDR corrected *p*-value = 3.79E-04). A most interesting finding was the response to hypoxia (Benjamini and Hochberg FDR corrected *p*-value = 3.31E-04). In general, hypoxia is important in drug resistance and poor survival [17]. Therefore, targeting hypoxia might be a practical way to improve patient survival rate of patients with astrocytoma and GBM [18].

Our KEGG pathway [10] analysis based on ToppFun [11] further highlighted a few important cancer-related signaling pathways, such as the PI3K-Akt signaling pathway (corrected *p*-value = 8.04E-05), pathways in cancer (corrected *p*-value = 5.32E-10), proteoglycans in cancer (corrected *p*-value = 3.33E-06), and the advanced glycation end products-receptor for advanced glycation end products pathway (corrected *p*-value = 1.201E-5). More interestingly, signaling by interleukins (corrected *p*-value = 3.7E-05) and cytokine signaling in the immune system (corrected *p*-value = 1.06E-03) highlighted the importance of interleukins in the progression of brain cancer. Previous observations confirmed that many cytokines (mainly interleukins) are involved in brain cancer aggressiveness and the generation of disease-associated pain [19]. In summary, all our functional analyses demonstrated that subtype-specific gene mining using the BCGene database may be used to identify common genes in different brain cancer subtypes and to explore potential common molecular mechanisms.

### Identify top-ranked genes with evidence mentioned only once in the literature

To further explore the curated genes’ relevancies to brain cancer, we ranked all the 1421 genes based on the 27 most reliable brain cancer genes as training set. The reliability of these 27 genes are based on each gene having 20 or more evidentiary mentions in the literature. This ranking result is to generate relatively importance to the remaining 1394 (1421 minus 27) genes in our database (Table S3). With similar functions to the 27 genes in the training set, the subsequent 100 top-ranked genes are likely important in brain cancer development. And within those top-ranked 100 genes, 33 were linked only by a single support from the literature. Thus, we consider that the roles of those 33 genes in brain cancer development are likely underestimated.

**Fig. 5:**
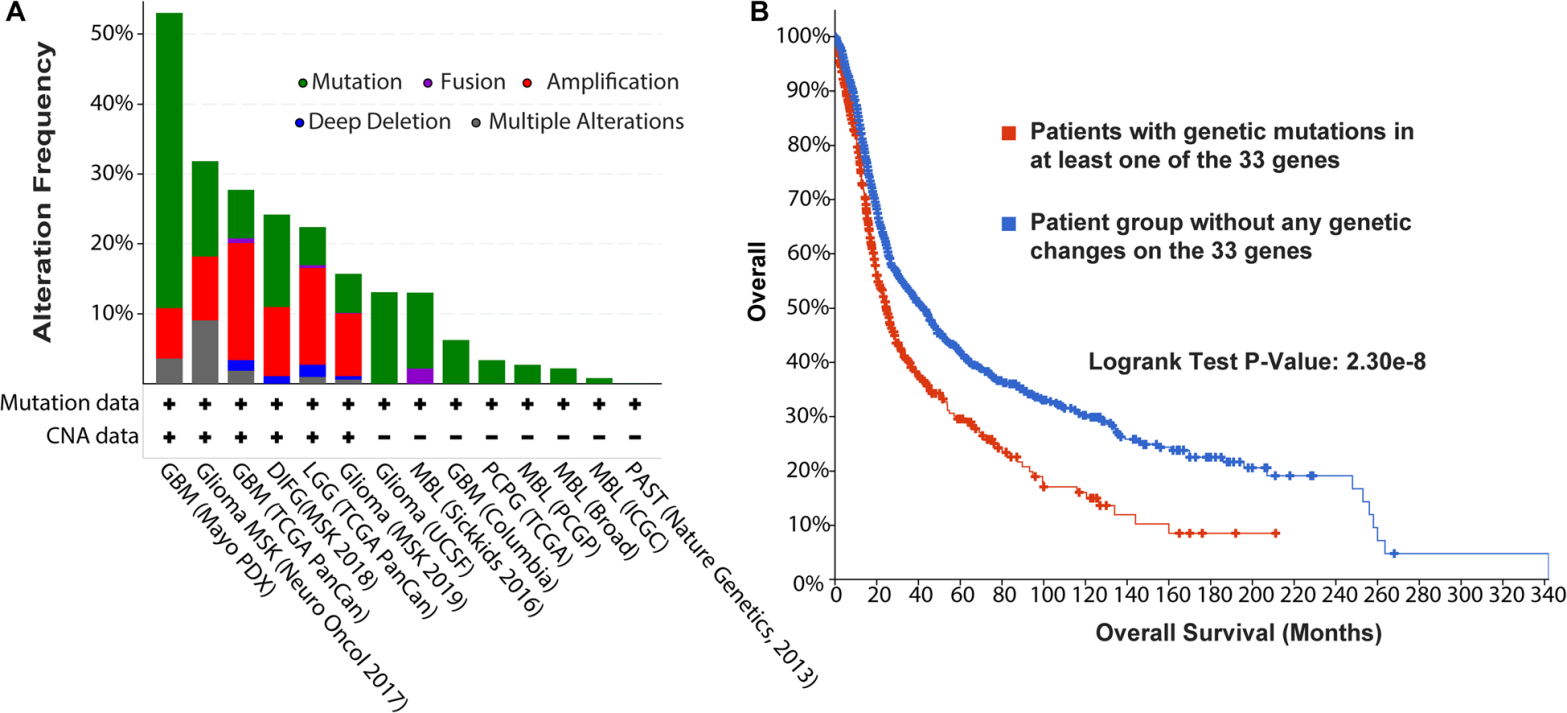
Mutation of the 33 top-ranked genes and overall patient survival. **A** The mutational frequency comparison of the 33 genes in 14 brain cancer studies. **B** The overall patient survival analysis of 2997 samples from the 14 studies. The dataset abbreviations are The Cancer Genome Atlas (TCGA), International Cancer Genome Consortium (ICGC), Glioblastoma (GBM), Memorial Sloan Kettering Institute (MSK), Desmoplastic Infantile Ganglioglioma (DIFG), Low Grade Glioma (LGG), Medulloblastoma (MBL), Pheochromocytoma and Paraganglioma (PCPG), Pilocytic Astrocytoma (PAST)

To investigate the potential oncogenic roles of those 33 genes, we used the large-scale cancer genomics datasets in cBioportal [12]. Altogether, we combined 2997 samples from 14 independent studies, including four datasets related to medulloblastoma, two datasets related to glioma, two GBM studies, two LGG studies, a study of anaplastic oligodendroglioma and anaplastic oligoastrocytoma, a study of a brain tumor patient-derived xenograft, an investigation of pilocytic astrocytoma, and a dataset of pheochromocytoma and paraganglioma. As shown in Figure S1, sample-based mutational patterns revealed 536 samples (18% of the total 2997 samples) that had at least one genetic mutation related to one of the 33 genes. After closely scrutinizing their subtype information (Fig. 5A), we found that the 33 genes were highly mutated in the glioma and GBM datasets but had relatively low mutational rates in the four datasets related to medulloblastoma. Interestingly, those 33 genes had a huge effect on patient survival (Fig. 5B). Among the 2303 patients with survival information, 467 of them had one or more genetic mutations in the 33 genes. The median survival of those 467 patients was 24.59 months, but the remaining 1836 patients’ median survival was 42.20 months, a very significant difference (log rank test, *p* = 2.30E-8).

**Fig. 6:**
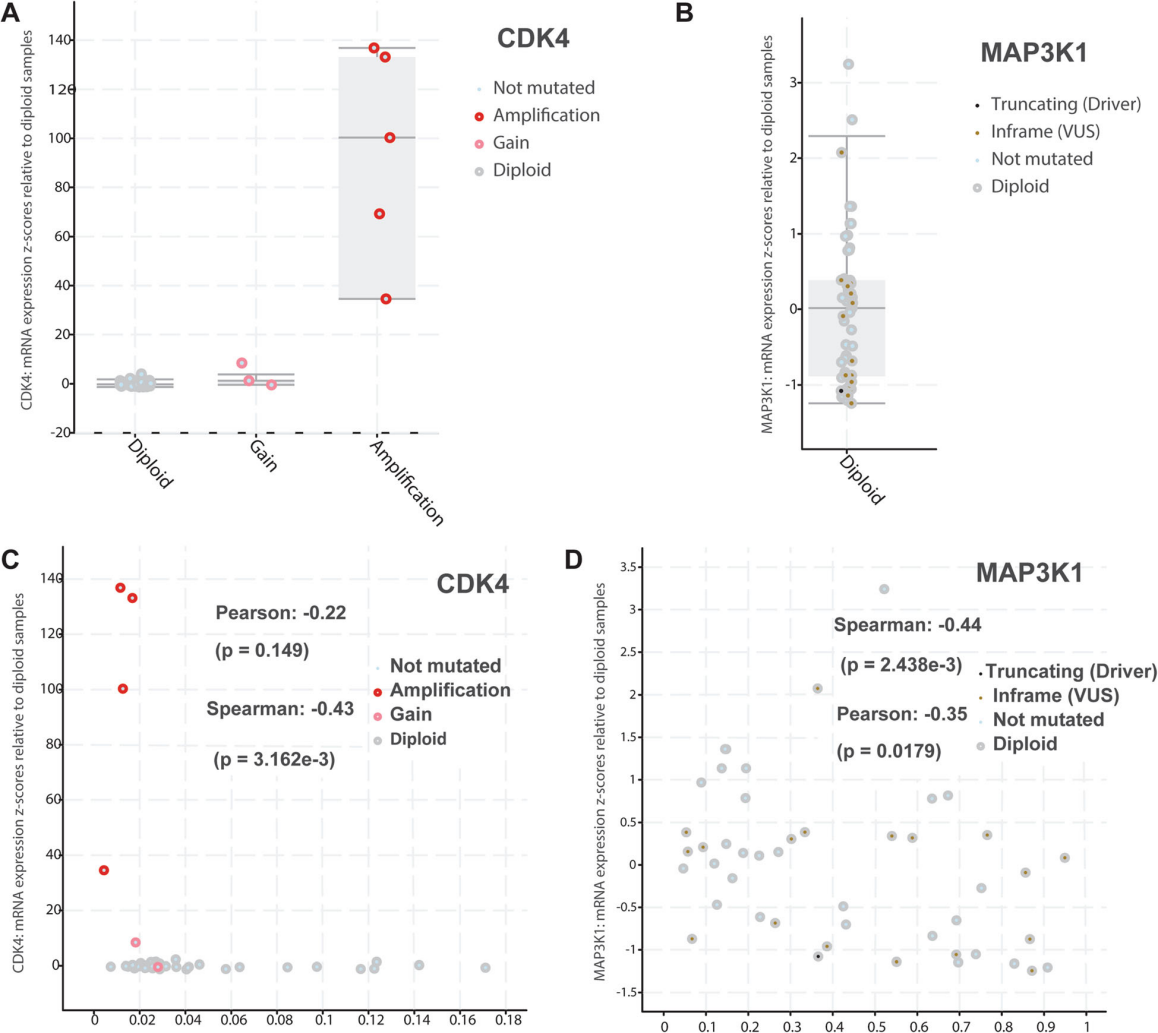
Correlation analyses for *CKD4* and *MAP 3 K1*. **A** and **B** The relationship between copy number changes and matched mRNA expression for *CDK4* (**A**) and *MAP 3 K1* (**B**). Data are means with standard errors bounded by the gray boxes and the whiskers are the 95% confidence interval. **C** and **D** The relationships between DNA methylation and matched mRNA expressions for *CDK4* (**C**) and for *MAP 3 K1* (**D**)

Among the 536 samples with genetic mutations in one or more of the 33 genes, the top-ranked gene, *CDK4*, was mutated in 202 samples (8% of the 2997 samples) and the second-ranked gene, *MAP 3 K1*, was mutated in 79 samples (2.8%), and 8 of those samples also had a *CDK4* mutation. Since the mutated genes in that mutational pattern are almost mutually exclusive, they may have complementary roles in the progression of brain cancer [20]. As shown in Fig. 6A, amplified *CDK4* in five samples coincided with mRNA up-regulation, but four of the five samples had low methylation, which could have caused the increased mRNA expression (Fig. 6C). However, *MAP 3 K1*’s correlation patterns were strikingly different than *CDK4*’s (Fig. 6B, D). Altogether, *CDK4* provides a good example of consistent mRNA up-regulation based on both amplification and methylation patterns, and *MAP 3 K1* may be a good candidate for evaluating some brain cancers’ progressions, but those possibilities need further study.

In summary, the functional similarity-based gene prioritization identified 33 top-ranked brain cancer-implicated genes with evidence mentioned only once in the literature. By focusing on 2997 samples from 14 independent brain cancer genetic datasets, we found that these 33 genes are highly mutated in hundreds of brain cancer samples and significantly associated with survival time. In addition, we found a mutually exclusive mutational pattern between the two top-ranked genes, *CDK4* and *MAP 3 K1*, which affected more than 200 brain cancer patients. Therefore, we consider that these two genes might be the most promising genes and might play important roles in the development of brain cancer.

## Conclusions

We have constructed a public repository, the brain cancer gene database (BCGene), which provides literature-based information for 1421 unique human genes by curating thousands of published articles. The main features of BCGene include: 1) manually curated literature; 2) cancer subtype information; 3) comprehensive function and annotation; 4) online-based data browsing system; 5) downloadable data for large-scale data integration. The database contains both microarray and in situ hybridization data, much of which is described here for the first time. Taken together, BCGene might significantly advance the understanding of genetics in brain cancer and provides a timely and valuable resource for the brain cancer genomics community. From our data collection, 809 gliomas, 450 lymphomas, and 354 neuroectodermal tumor-related genes are supported by evidence in the literature. This comprehensive data collection not only presents the genetic heterogeneity of brain cancer, but also provides comparable genetic resources for exploring the common genetic mechanisms among different brain cancer subtypes. Our future plans are to focus on the subtype-unique gene sets, which may both aid the understanding of underlying disease mechanisms and identify novel therapies for specific brain cancer subtypes.

## Supplementary Information

**Additional file 1: Fig. S1** The top ranked genes’ sample-based mutational profiles across 2997 patient samples from 14 different brain cancer studies.

**Additional file 2: Table S1.** General information of the 1421 human brain cancer genes in the BCGene.

**Additional file 3: Table S2.** Curated brain cancer subtype information.

**Additional file 4: Table S3.** Gene prioritization results for all the 1394 genes with less than 20 evidentiary mentions in the literature.

## Data Availability

The data-sets generated and/or analysed during the current study are available in the BCGene repository, http://soft.bioinfo-minzhao.org/bcgene/. The data-sets used and/or analysed during the current study available from the corresponding author on reasonable request. All data generated or analysed during this study are included in this published article and its supplementary information files.

## Abbreviations

BCGene: Brain Cancer-implicated Genes and Literature database
GBM: Glioblastoma
LGG: Low Grade Gliomas
TCGA: The Cancer Genome Atlas
GeneRIF: Gene Reference Into Function
ICGC: International Cancer Genome Consortium
MSK: Memorial Sloan Kettering Institute
DIFG: Desmoplastic Infantile Ganglioglioma
MBL: Medulloblastoma
PCPG: Pheochromocytoma and Paraganglioma
PAST: Pilocytic Astrocytoma
