# Supplementary figures and images for: Online database for brain cancer-implicated genes: exploring the subtype-specific mechanisms of brain cancer
Source: BMC Genomics. 2021 Jun 18;22:458. doi: 10.1186/s12864-021-07793-x (PMC8214279; doi:10.1186/s12864-021-07793-x)

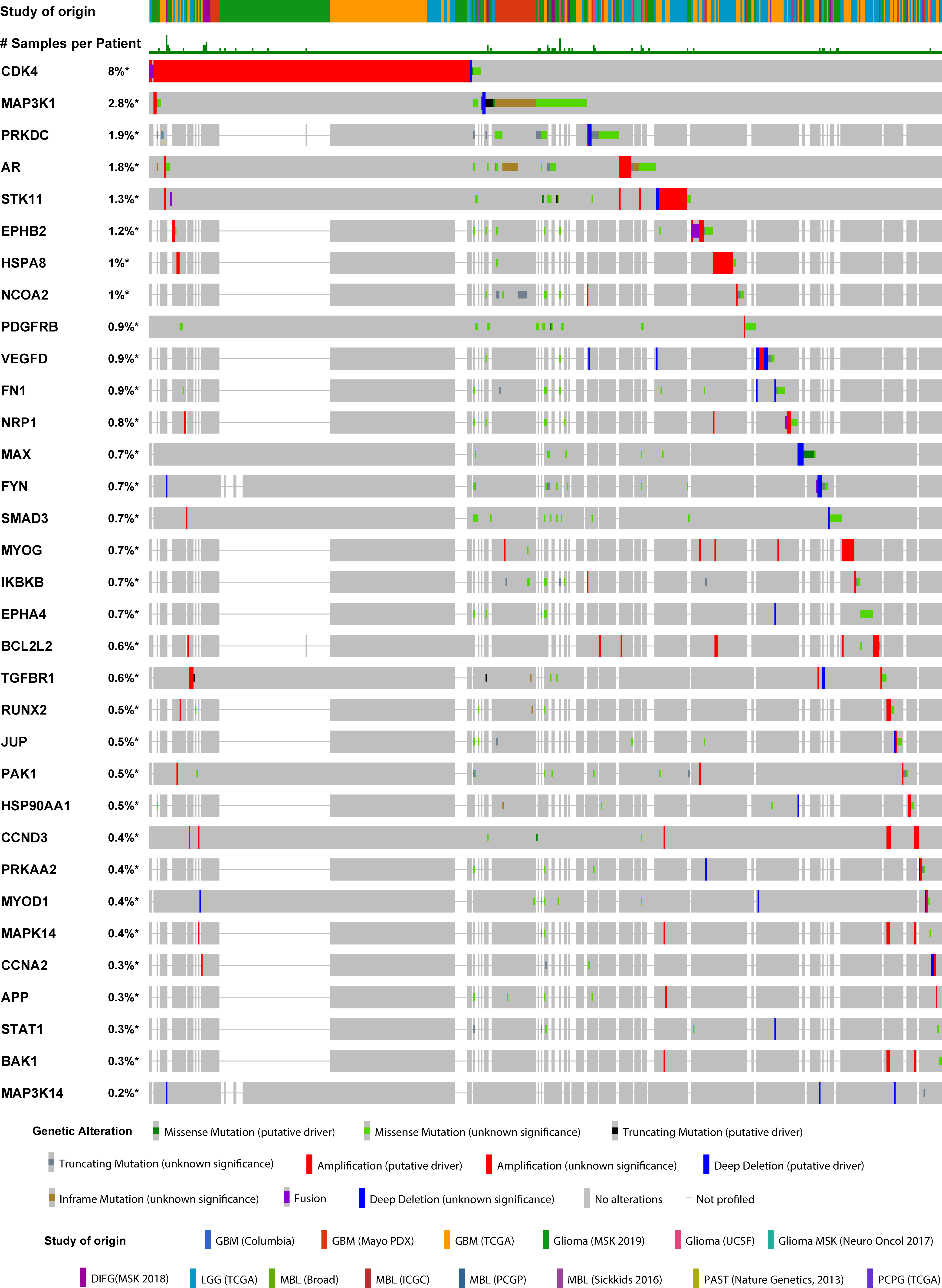

Supplement: Supplementary file 1 — Additional file 1: Fig. S1 The top ranked genes’ sample-based mutational profiles across 2997 patient samples from 14 different brain cancer studies. [file 12864_2021_7793_MOESM1_ESM.tif]
